# Ingestion of spinosad-containing toxic sugar bait alters *Aedes albopictus* vector competence and vectorial capacity for dengue virus

**DOI:** 10.3389/fmicb.2022.933482

**Published:** 2022-08-26

**Authors:** Abdullah A. Alomar, Bradley H. Eastmond, Zoi Rapti, Edward D. Walker, Barry W. Alto

**Affiliations:** ^1^Florida Medical Entomology Laboratory, University of Florida, Vero Beach, FL, United States; ^2^Carl R. Woese Institute for Genomic Biology, University of Illinois at Urbana-Champaign, Urbana, IL, United States; ^3^Department of Mathematics, University of Illinois at Urbana-Champaign, Urbana, IL, United States; ^4^Department of Entomology, Michigan State University, East Lansing, MI, United States; ^5^Department of Microbiology and Molecular Genetics, Michigan State University, East Lansing, MI, United States

**Keywords:** dengue virus, toxic sugar bait, viral infection, vectorial capacity, survival, *Aedes albopictus*

## Abstract

Dengue virus (DENV) is a highly prevalent vector-borne virus that causes life-threatening illnesses to humans worldwide. The development of a tool to control vector populations has the potential to reduce the burden of DENV. Toxic sugar bait (TSB) provides a form of vector control that takes advantage of the sugar-feeding behavior of adult mosquitoes. However, studies on the effect of ingestion of toxins in TSB on vector competence and vectorial capacity for viruses are lacking. This study evaluated vector competence for DENV serotype-1 of *Aedes albopictus* at 7 and 14 days post-ingestion of TSB formulated with spinosad (of bacteria origin) as an oral toxin. Our results and others were modeled to estimate effects on *Ae. albopictus* vectorial capacity for DENV. Ingestion of TSB caused a reduction in survival of females, but increased mosquito susceptibility to DENV infection, disseminated infection, and transmission. However, this increase in vector competence was obviated by the reduction in survival, leading to a lower predicted vectorial capacity. The findings of this study highlight the importance of evaluating the net impact of TSB ingestion on epidemiological parameters of vectorial capacity in the context of vector control efforts to reduce the risk of transmission of vector-borne viruses.

## Introduction

Dengue viruses (DENVs), encompassing four serotypes, are major causes of morbidity and mortality in humans, with millions of cases recorded annually in tropical and sub-tropical regions (Balmaseda et al., 2006; Vasilakis et al., 2010; Gebhard et al., 2011; Bhatt et al., 2013). These viruses are enveloped, (+)-sense, single-stranded RNA genomes and are primarily transmitted amongst humans by *Aedes aegypti* mosquitoes. However, *Ae. albopictus* serves as a vector for DENV and chikungunya virus (CHIKV) under many circumstances, such as in areas where *Ae. aegypti* is less abundant or absent (Effler et al., 2005; Charrel et al., 2007; Ratsitorahina et al., 2008; Leroy et al., 2009; Christofferson, 2015). *Aedes albopictus* is an invasive mosquito species that has a wide global distribution, native to the tropics of Southeast Asia (Paupy et al., 2009). This species poses a significant public health concern because of its ability to proliferate in human habitats and its competence for transmission of DENV and other emerging viruses, such as Zika virus (ZIKV) and CHIKV (Gratz, 2004; Calle-Tobón et al., 2020). The capability of *Ae. albopictus* to serve as a vector of pathogens has been experimentally demonstrated for numerous viruses belonging to the genera *Flavivirus*, *Alphavirus*, and *Bunyavirus* (Gratz, 2004).

Lowering the public health burden and risk of emerging viruses has most often been achieved with the application of chemical substances, such as insecticides. The application of insecticide results in either reduction of the population size of the main virus-carrying vectors, the reduction of the average age in the vector population, or both (Catano-Lopez et al., 2019). Targeted delivery of toxicants takes advantage of the inherent resource-acquiring behaviors of mosquitoes. An example is the sugar-feeding behavior of mosquitoes, which meets energy-demanding activities including flight, host-seeking, mating, blood-feeding, and reproduction (Foster, 1995; Fikrig et al., 2020). It further contributes to components of vectorial capacity, especially survival (Stone and Foster, 2013). Mosquitoes obtain sugar to meet this dietary need from several sources, including floral or extrafloral nectaries, rotting or damaged fruit, and honeydew (Foster, 1995; Fikrig et al., 2020). Toxic sugar bait (TSB) exploits mosquito physiological needs for sugar to deliver gut toxins through ingestion of toxic substances incorporated into the sugar source (Fiorenzano et al., 2017). The application of TSB containing different active toxins under both laboratory and field conditions has proven effective in reducing the density of diverse mosquito species following oral ingestion (Fiorenzano et al., 2017). For example, higher mortalities in adults of *Ae. aegypti*, *Culex quinquefasciatus*, and *Ae. albopictus* were observed following the ingestion of *Bacillus thuringiensis israelensis* sugar bait in comparison to adults that ingested sugar bait without *Bacillus thuringiensis israelensis* (Davis et al., 2021). The residue from spraying of boric acid sugar bait on plant surfaces provided an effective control against *Ae. albopictus*, *Ochlerotatus taeniorhynchus*, and *Cx. nigripalpus* upon oral ingestion (Xue et al., 2006; Müller et al., 2010b). The application of TSB with different active ingredients onto surfaces in natural habitats led to population declines of *Anopheles gambiae*, *An. sergentii*, and *An. claviger* (Müller and Schlein 2008; Müller et al., 2008, 2010a). However, the effects of TSB on parameters of vectorial capacity aside from population density, such as vector competence and survivorship, in particular, are poorly known.

The objective of this study was to assess the effects of ingestion of TSB on *Ae. albopictus* vector competence, survival, and vectorial capacity for DENV serotype-1. Here, we utilized spinosad as an oral ingestible toxin in TSB formulation. Spinosad is an insecticide consisting of secondary fermentation metabolites of the actinomycete soil bacterium, *Saccharopolyspora spinosa* (Kirst, 2010). Spinosad has a favorable mammalian and environmental toxicological profile and can be used as an oral toxin against numerous mosquito species (Müller et al., 2008; Müller and Schlein, 2008; Alomar et al., 2022).

## Materials and methods

### Mosquitoes

Larvae were collected from a population of *Ae. albopictus* in Vero Beach, Florida, and a laboratory colony was established. Mosquitoes were in the F4 generation when used. Maintenance of the colony was described elsewhere (Alomar and Alto, 2021, 2022a).

### Toxic sugar bait preparation

A 1% spinosad (Southern Ag, Palmetto, FL, United States) was incorporated in a 10% sugar solution to prepare the TSB formulation as described elsewhere (Alomar et al., 2022). From this stock solution, dilutions were prepared to determine its oral acute toxicity to female *Ae. albopictus* using concentrations of 1, 3, 5, 7, 10, 12, 15, 18, 22, and 35 ppm. Females were deprived of access to sugar for 24 h, then placed in cages and allowed to ingest different concentrations of TSB via cotton pledgets. Each concentration was replicated three times, each consisting of 25 females. Controls were allowed to ingest only 10% SB without the toxin. Mosquitoes were held at 28°C and acute mortality was recorded at 24 h. The median lethal concentration of TSB was determined by probit analysis, and used in the following experiments.

### Dengue virus propagation and mosquito infection

The DENV serotype-1 (strain BOLKW010, GenBank: JQ675358.1) used in this study was originally isolated from a DENV-infected patient in Key West, FL, United States, in 2010 and provided by the Florida Department of Health Bureau of Public Health Laboratories. Propagation of DENV was conducted in kidney epithelial cells of African green monkey *Cercopithecus aethiops* according to standard conditions (Alomar et al., 2020). We passaged DENV three times in cell culture before use in the infection study. The primate cell line (Vero E6, ATCC CRL-1586) was cultured in growth media (Medium 199, Cytiva HyClone), supplemented with 10% heat-inactivated fetal bovine serum, penicillin–streptomycin, and mycostatin and maintained at 37°C in a 5% CO_2_ atmosphere. Confluent monolayers of cells were inoculated at a multiplicity of infection of 0.01 and incubated at 37°C in a 5% CO_2_ atmosphere for 1 h. After this incubation period, 24 ml of medium 199 were added to the cells propagated in culture flasks, and further incubated for 7 days. To prepare an infectious bloodmeal, defibrinated bovine blood (Hemostat Laboratories, Dixon, CA, United States) and adenosine triphosphate as a phagostimulant (Amersham Biosciences, Piscataway, NJ, United States) were combined with freshly harvested media from DENV-infected cells (Alomar and Alto, 2022b).

**Figure 1:**
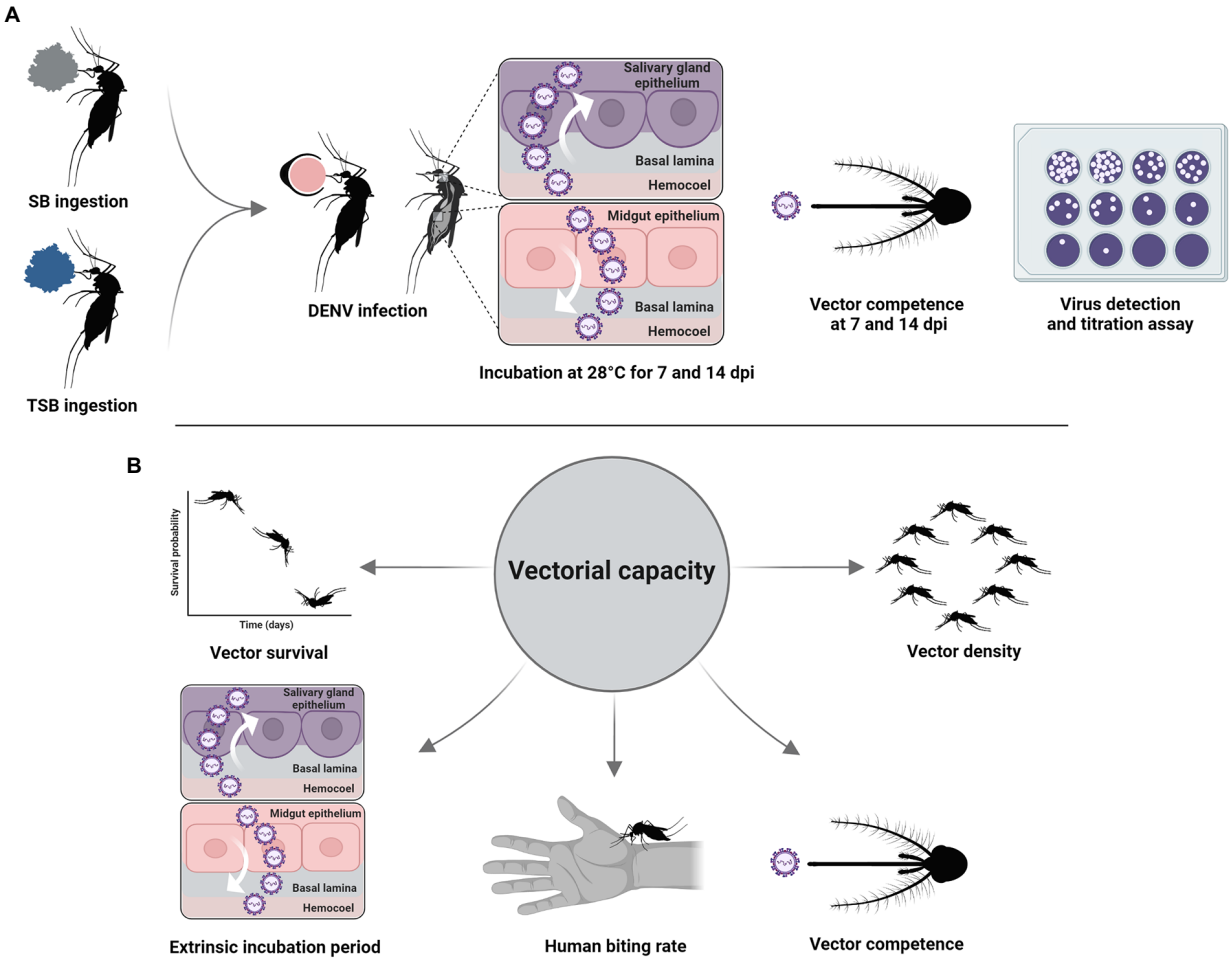
Experimental workflow **(A)**. Vectorial capacity parameters **(B)**.

Surviving 8-to 10-day-old females that had fed on a median lethal concentration of TSB 4 days previously were offered DENV-infected blood through 37°C pre-heated Hemotek feeders (Discovery Workshops, Accrington, United Kingdom) for 1 h. Control mosquitoes were handled similarly but were not exposed to TSB (Figure 1). Under CO_2_ anesthesia, fully blood-engorged females were transferred to new clean cages, revived, and incubated at 28°C on a 12:12-h light: dark photoperiod regimen. Partially blood-engorged and unfed females were discarded. At 7-and 14-day post-infection (dpi), mosquitoes were dissected to remove legs and wings from bodies using sterile forceps. Dissected bodies and legs were placed into separate centrifuge tubes (Thermo Fisher Scientific, Waltham, MA, United States) containing 1 ml medium 199 and stored at −80°C. At each time point, the saliva of females was collected using a forced salivation method (Alomar et al., 2020). Briefly, wings and legs were removed, and proboscises inserted for 45 min into microhematocrit glass capillaries (Thermo Fisher Scientific, Waltham, MA, United States) holding type B immersion oil (Cargille Laboratories, Cedar Grove, NJ, United States). After this time interval, saliva within capillaries was dispensed into separate centrifuge tubes (Thermo Fisher Scientific, Waltham, MA, United States) containing 300 μl medium 199 and stored at −80°C until processing.

### Dengue virus detection and titration assay *in vitro*

The presence and viral titers of DENV infectious particles in mosquito tissues were determined using plaque assays with primate cells. The day prior to the assays, 12-well plates were seeded with primate cells (1.5 × 10^5^ cells/ml) and maintained with medium 199, supplemented with 10% heat-inactivated fetal bovine serum, penicillin–streptomycin, and mycostatin, at 37°C and a 5% CO_2_ atmosphere. When cells reached confluency, mosquito tissues were homogenized at 19.5 Hz for 3 min, centrifuged at 13,200 rpm for 5 min, 10-fold serially diluted, and 200 μl of diluent from dilutions were inoculated onto cell monolayers after the removal of medium 199. Then, plates were incubated at 37°C in a 5% CO_2_ atmosphere for 1 h and rocked every 15 min to allow for viral adsorption to cells. After this interval, an immobilizing overlay of 1% methylcellulose was applied to the wells (1.5 ml/well) to restrict DENV growth to the originally infected foci of cells, followed by incubation for 7 days. After this period, methylcellulose overlays were removed, and cells were stained with crystal violet 0.25% hexamethyl pararosaniline chloride solution (1 ml/well) to enhance visualization and enumeration of viral plaques. Each well was classified as virus-positive or virus-negative based on the respective presence or absence of DENV viral plaques. Titrations of DENV in mosquito tissues were determined by counting the number of viral plaques formed in each well, expressed as plaque forming Units per milliliter (PFU/ml). Susceptibility to DENV infection was calculated as the percent of mosquitoes with the presence of DENV infectious particles in their bodies among engorged mosquitoes. Disseminated infection and transmission of DENV were calculated as the percent of mosquitoes with the presence of DENV infectious particles in their legs and saliva, respectively, among engorged mosquitoes.

### Adult survival

To assess the effects of TSB ingestion on adult survival, 2-to 4-day-old females were allowed to ingest a median lethal concentration of TSB determined from the above experiment. Blue food coloring was added to the TSB formulation, providing a visual marker of ingestion. Those mosquitoes with a stretched, blue abdomen were transferred to new cages with constant access to SB. Controls not given TSB were handled similarly. Adult cages were held at 28°C and mortality was scored daily. Five replicates (*n* = 50/replicate) were performed for treatment and control groups.

### Analytical model of vector competence and vectorial capacity

To analyze the effects of TSB ingestion on the vectorial capacity for DENV, we used the cumulative vectorial capacity (*cVC*) equation developed by Christofferson and Mores (2011). The *cVC* is informed by a quantity termed effective vector competence (*evc*), instead of a fixed value, using the following regression model for the predicted vector competence (*b*) on day n post ingestion of virus in a blood meal:


(1) $$b\left(n\right)=\beta_{1}n+\beta_{0}$$

where $\beta_{1}$ is the proportion of the population competent to transmit per day of life, and $\beta_{0}$ is the y-intercept. Effective vector competence (φ) is then defined as:


(2) $$evc=\varphi =\overset{z}{\underset{t}{\int }}p^{n}\left(\beta_{1}n+\beta_{0}\right)dn$$

The interval over which this line is constructed has lower-and upper-time limits, t and z (here, values *t* = 7 and z = 14 days). The values for the extrinsic incubation period were within the expected range for DENV (Chan and Johansson, 2012). Effective vector competence is inserted into the *cVC* equation as:


(3) $$cVC=\frac{ma^{2}\varphi }{-\ln\left(p\right)}$$

We estimated parameters for the model from this study and literature sources listed in Table 1. From these equations, we analyzed the impacts of TSB ingestion on *cVC* using our daily survival probability data (p = 0.42) for TSB and (p = 0.94) for SB (control), assuming equal daily survival probability (p = 0.94) between TSB and SB treatments, or assuming higher daily survival probability (p= 0.94) for TSB and lower daily survival probability (p = 0.42) for SB, across a range of human biting rates. We then estimated the impacts of TSB ingestion on *cVC* across a range of survival probabilities while holding mosquito density *m* (mosquitoes/person) constant (*m* = 10; Carrieri et al., 2012; Oki and Yamamoto, 2013) and human biting habit *a* (proportion of mosquitoes that blood-fed on humans) constant (*a* = 0.79–0.96; Valerio et al., 2010).

**Table 1:** Vectorial capacity model parameters. Vector density (*m*), human biting rate (*a*), daily survival probability (*p*), vector competence (*b*), pathogen incubation period (*n*).

Parameters	Value	Source
*m*	10	Carrieri et al. (2012), Oki and Yamamoto (2013)
*a*	0.79–0.96	Valerio et al. (2010)
*p*	0.42 (TSB)/0.94 (control)	This study
*b*	0.00–0.57	This study
*n*	7–14 days	This study

### Data analysis

TSB concentration-acute mortality data were subjected to probit analysis (Finney, 1952) to estimate the median lethal concentration. The effects of treatment and time and their interaction on mosquito vector competence for DENV were assessed using logistic regression analysis. Following the detection of significant effects, a pairwise comparison was conducted using the Tukey–Kramer test. Student’s *t*-tests were used to compare DENV titers on mosquito tissues between treatments. A Cox proportional hazards regression model was used to analyze adult survival following ingestion of TSB. The differences between treatments were considered to be statistically significant at *p* < 0.05. All analyses were run using SAS version 9.22 (SAS Institute Inc., Cary, North Carolina, Uinted States).

## Results

### Median lethal concentration and vector competence estimation

Probit analysis showed that ingestion of TSB induced acute mortality among females of *Ae. albopictus* with an estimated median lethal concentration of 9.1 ppm. To evaluate *Ae. albopictus* vector competence for DENV following the TSB ingestion (treatment), a total of 135 female mosquitoes were examined for susceptibility to DENV infection, disseminated infection, and transmission at two time points (7 and 14 dpi). The titer of DENV in the infectious bloodmeal was 6.1 log_10_ PFU/ml. Logistic regression analysis revealed significant effect of treatment on infection (*χ^2^* = 8.16, *p* = 0.004), disseminated infection (*χ^2^* = 13.53, *p* = 0.0002), and transmission (*χ^2^* = 8.52, *p* = 0.003), but not time or their interaction.

**Figure 2:**
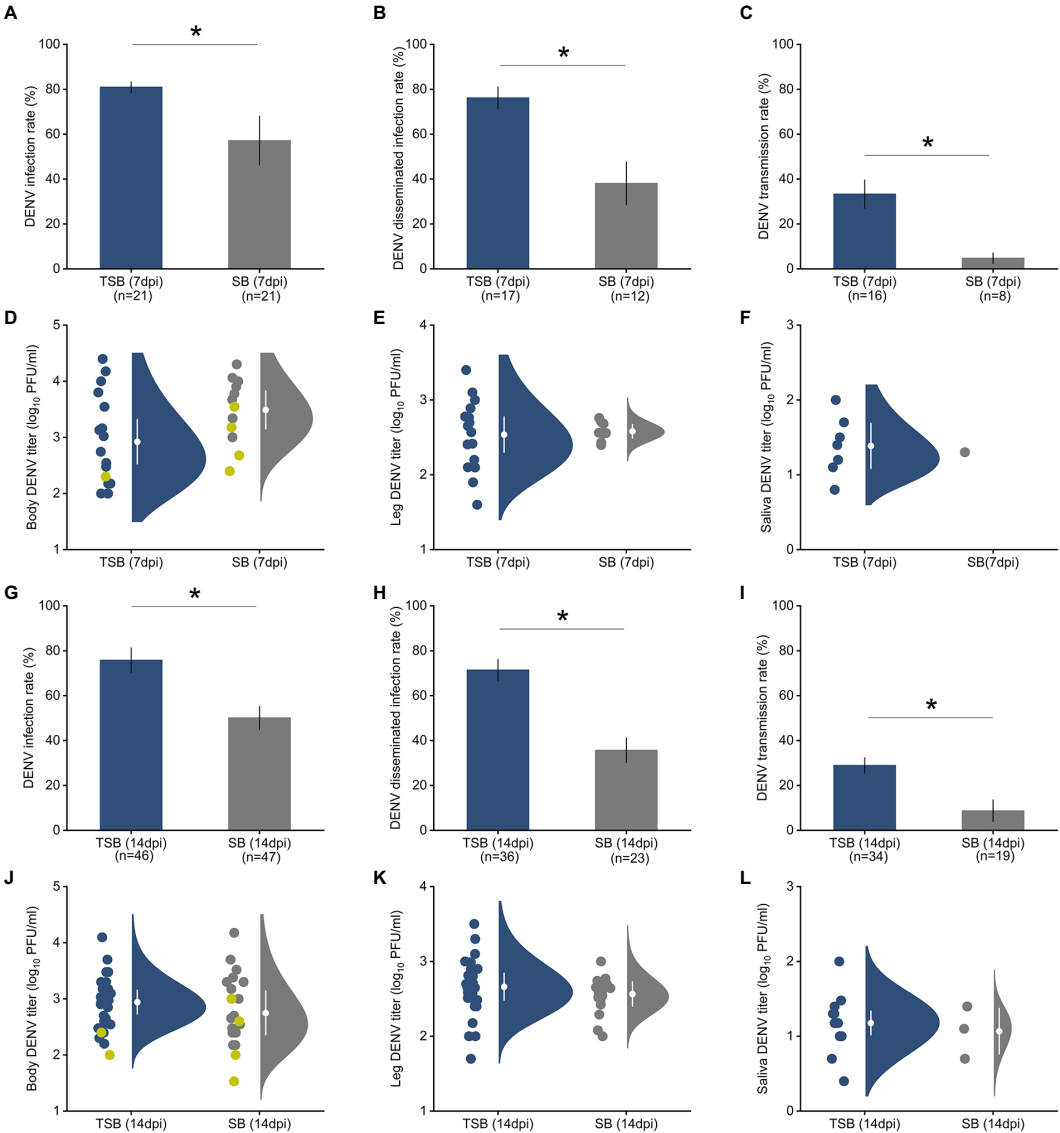
Ingestion of TSB effects on *Ae. albopictus* susceptibility to DENV infection **(A)**, disseminated infection **(B)**, and transmission **(C)** rates at 7 dpi. The bars represent the means and standard error of means. Raincloud plots represent distributions of DENV titers as measured by plaque assay in mosquito tissues, body **(D)**, legs **(E)**, and saliva **(F)** at 7 dpi. Susceptibility to DENV infection **(G)**, disseminated infection **(H)**, and transmission **(I)** at 14 dpi. Raincloud plots represent distributions of DENV titers in mosquito tissues, body **(J)**, legs **(K)**, and saliva **(L)** at 14 dpi. Each circle indicates a data point for an individual DENV-infected female. Yellow circles in panels **(D,J)** represent viral titer in mosquito bodies with non-disseminated infection, and gray and navy blue circles represent mosquito bodies with disseminated infections. White circles and lines inside the rainclouds represent the means and standard error of means, respectively. The density distribution of data is indicated by the filled area. We observed a significant increase in susceptibility to DENV infection, disseminated infection, and transmission after TSB ingestion at both time points (7 and 14 dpi). However, DENV titers in mosquito tissues (body, legs, saliva) were not affected by TSB ingestion at the two time points. The significant effects of TSB ingestion on DENV infection measurements were determined by logistic regression analysis with *post hoc* pairwise comparison using the Tukey–Kramer test. The number of mosquitoes assayed is indicated below each bar. Asterisks (*) on the top of the bars represent *p* < 0.05.

*A. albopictus* females that ingested TSB were 1.5 times more susceptible to DENV infection than mosquitoes feeding on SB lacking the toxin (controls; Figures 2A,G). Similarly, *Ae. albopictus* females that ingested TSB were nearly twice as likely to have disseminated infection, an advanced state of infection, relative to controls (Figures 2B,H). Transmission, as measured by saliva infection, was 3.5 times higher in females that ingested TSB relative to controls (Figures 2C,I).

Student’s *t*-tests showed that ingestion of TSB had no effect on DENV titers at 7 dpi in the mosquito’s body (*t* = 0.49, *p* = 0.65, Figure 2D), legs (*t* = −0.98, *p* = 0.38, Figure 2E), and saliva (*t* = −1.37, *p* = 0.24, Figure 2F) in comparison to the control. There was also no significant effect of TSB ingestion on DENV titers in the body (*t* = −0.23, *p* = 0.83, Figure 2J), legs (*t* = −1.22, *p* = 0.29, Figure 2K), and saliva (*t* = −1.32, *p* = 0.25, Figure 2L) at 14 dpi.

### Adult survival

**Figure 3:**
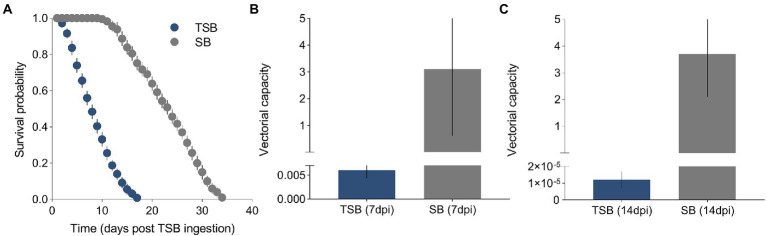
Survival curves of TSB-ingested mosquitoes and SB-ingested mosquitoes **(A)**. Vectorial capacity of TSB-ingested mosquitoes and SB-ingested mosquitoes at 7 dpi **(B)** and 14 dpi **(C)**. The data shown represent the means and standard error of means.

A total of 500 female mosquitoes were used to measure the effect of TSB ingestion on their duration of life. A Cox proportional hazards regression model showed that the survival of females was significantly affected by the ingestion of TSB (*χ^2^* = 2847.1, *p* = <0.0001, Figure 3A). The survival curves were different between treatments, with survival being heavily impeded in females that ingested TSB when compared to females that ingested SB (controls). The daily survival probability of adults was estimated as 0.42 and 0.94 for TSB-ingested mosquitoes and SB-ingested mosquitoes, respectively.

### Analytical model of vector competence and vectorial capacity

**Figure 4:**
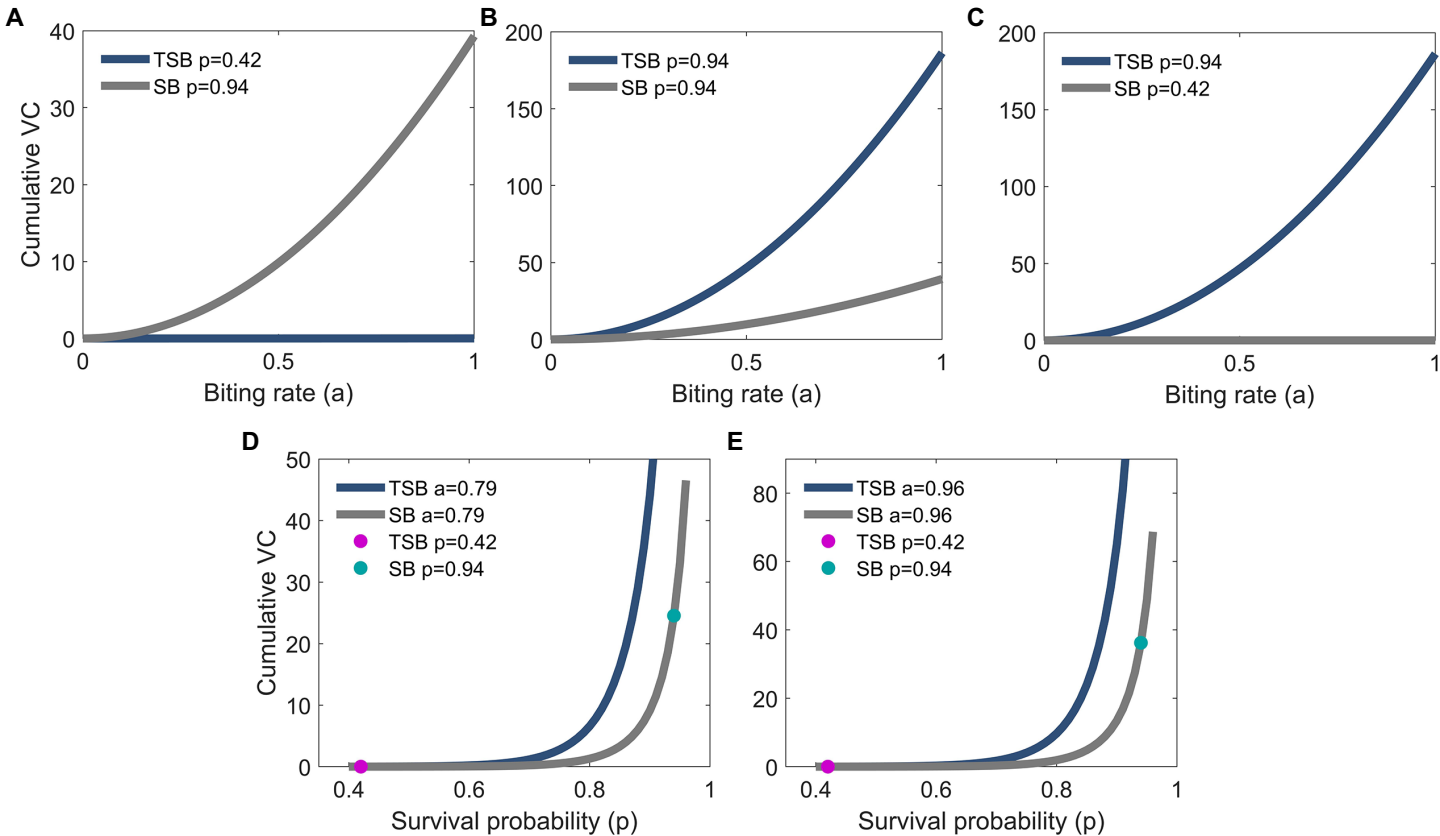
Biting rate effect on cumulative vectorial capacity (*cVC*) of TSB-ingested mosquitoes (navy blue line) and of SB-ingested mosquitoes (control; gray line). *cVC* is higher for control mosquitoes for the empirically observed survival probabilities in our study **(A)**, but the opposite is predicted assuming equal **(B)** or higher survival probabilities in the TSB-ingested mosquitoes **(C)**. *cVC* is evaluated using Eq. (3) with mosquito density set at *m* = 10 and the empirically obtained values of vector competence. The values for *cVC* in **(A)** are on a different scale than in panels **(B,C)**. Survival probability effect on *cVC* of TSB-ingested mosquitoes and control mosquitoes **(D,E)**. Empirical results are shown in dots (magenta for TSB and green for SB). *cVC* is evaluated using Eq. (3) with mosquito density set at *m* = 10 and the empirically obtained values of vector competence. The values for *cVC* in **(D)** are on a different scale than in **(E)**.

Models showed that *VC* was reduced in females that ingested TSB in comparison to females in controls (Figures 3B,C). Lower daily survival probability in TSB-ingested females reduced their *cVC* (Figure 4A). Assuming an equal daily survival probability for females in both treatments, or higher survival for TSB resulted in an increase of *cVC* in TSB-ingested individuals compared to SB-ingested mosquitoes (controls) (Figures 4B,C). Secondly, variations in daily survival probability had more notable impacts on *cVC* than variations in human biting rates (Figures 4D,E). This suggests that daily survival probability has a profound effect on *cVC*.

**Figure 5:**
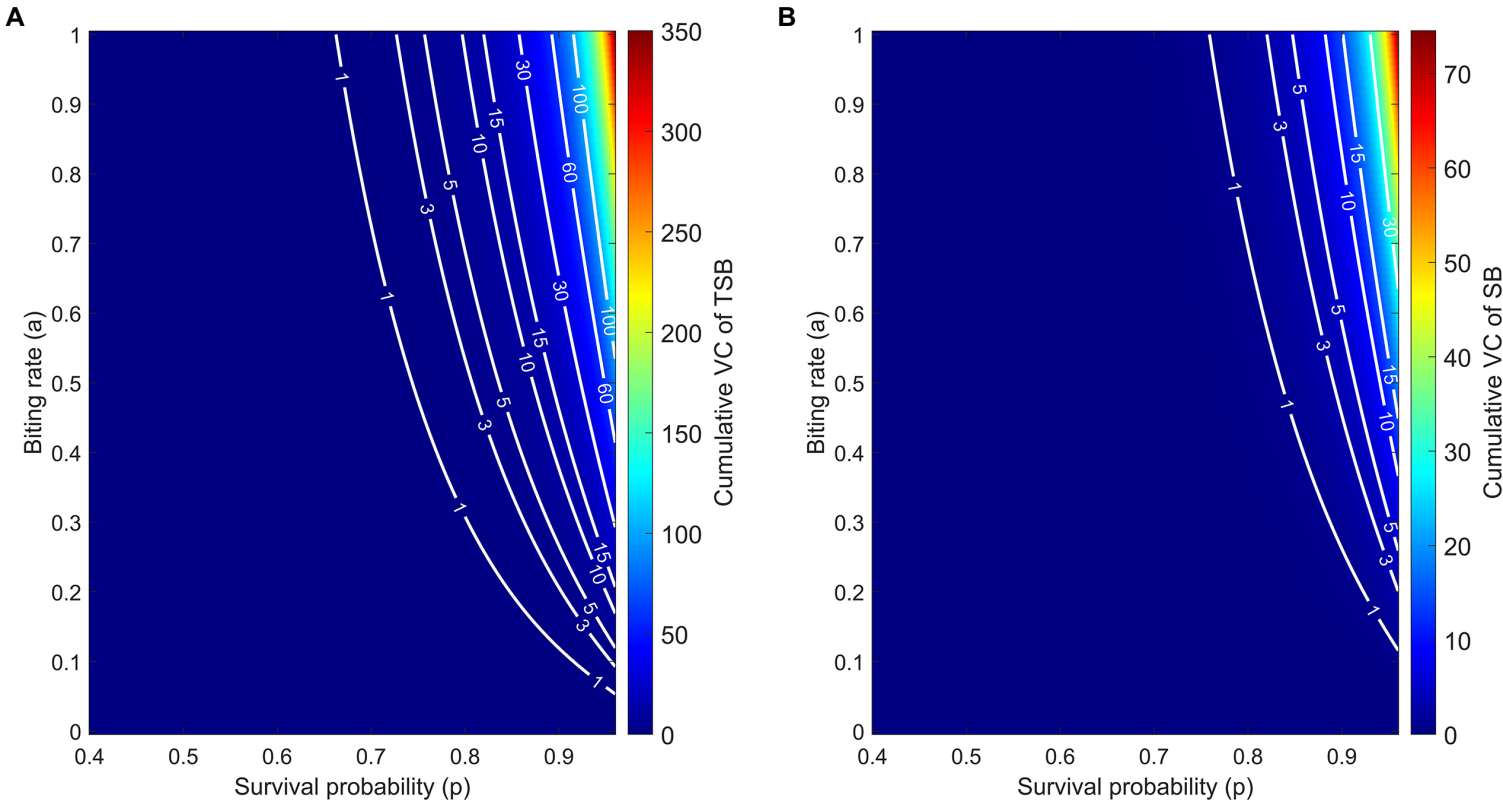
Cumulative vectorial capacity (*cVC*) as a function of both the daily survival probability (horizontal axis) and the biting rate (vertical axis) of TSB-ingested mosquitoes **(A)** and SB-ingested mosquitoes (control) **(B)**. Along the level curves (white curves) *cVC* assumes the annotated values, ranging from 0 to 350.1 for TSB-ingested mosquitoes and from 0 to 74.55 for control mosquitoes. *cVC* is evaluated using Eq. (3) with mosquito density set at *m* = 10 and the empirically obtained values of vector competence.

The model predicts that, due to the empirically observed higher vector competence of TSB-ingested mosquitoes, the *cVC* is higher compared to control mosquitoes. This feature persists over the wide range of biologically relevant values of biting rates and daily survival probabilities (Figures 5A,B). By comparing level curves, namely the white curves of constant *cVC* values, one observes in Figure 5 that to achieve the same value of *cVC*, control mosquitoes must have either larger survival probabilities or larger biting rates.

## Discussion

This study examined the effects of oral ingestion of TSB on female survival and vector competence for DENV in *Ae. albopictus*, and incorporated experimental findings into a statistical model assessing these effects on vectorial capacity. The results were paradoxical: whilst ingestion of TSB at a pre-determined median lethal concentration caused a reduction in the probability of daily survival among female mosquitoes surviving acute mortality (thereby having a negative effect on vectorial capacity), the susceptibility to DENV infection in those survivors, as measured by disseminated infection and transmission, increased. However, the net effect of these two outcomes was that ingestion of TSB lowered vectorial capacity because of the strong reduction effect in daily survival probability. These findings provide insight into the sublethal effects of TSB application in vector control.

A growing body of literature has shown that the application of TSB can effectively suppress the targeted mosquito population upon the ingestion of the oral toxin (Fiorenzano et al., 2017). In addition to the acute mortality by TSB ingestion, alterations in adult traits important for pathogen transmission were observed among surviving individuals. For instance, oral ingestion of TSB incorporating boric acid (a gut toxin) reduced adult mosquito daily survival probability, host-seeking, blood-feeding rates, fecundity, and fertility in *Ae. albopictus* (Ali et al., 2006; Wang et al., 2017). Consistent with these studies, we found that females who ingested TSB had substantially shorter daily survival than individuals in the control group. This reduction of vector survival may cause a profound effect in altering the vectorial capacity. Females may not survive long enough for an acquired infection to disseminate beyond the midgut and infect the salivary glands with the possibility for transmission to humans by bite. Our findings, along with others, suggest that TSB ingestion may induce latent effects (e.g., impair longevity) on survivors which would enhance the feasibility and efficacy of the TSB method in vector control.

A limitation of the vector competence component of this study was a modest sample size. This is in part attributable to mortality due to deliberately allowing mosquitoes to feed on a median lethal concentration of the TSB formulation. However, consistent directional effects and low variation in response variables, especially for day 14, suggest a strong relationship between ingestion of TSB and altered vector competence. To our knowledge, the current study represents one of the first assessments of the influence of TSB ingestion on the vector competence of mosquitoes for viruses and pathogen risk assessment. It is well known that adult mosquito responses to viral infection can be altered by biotic and abiotic factors, including exposure to insecticides, although the physiological mechanisms involved are not well understood (Alto and Lounibos, 2013). Adults of *Ae. aegypti* exposed to toxins of *Bacillus thuringiensis,* serovariety *israelensis,* as larvae developed higher DENV-1, DENV-2, and ZIKV titers of infection and had higher rates of disseminated infection (Moltini-Conclois et al., 2018; Carvalho et al., 2021). By contrast, another study showed no effect of exposure to these toxins during larval stages on susceptibility to DENV infection and disseminated infection in adult *Ae. aegypti* (Alto and Lord, 2016). Higher Sindbis virus dissemination rates were observed in adult *Ae. aegypti* exposed to malathion as larvae (Muturi et al., 2011). Exposure of larval *Ae. aegypti* to juvenile hormone analogs enhanced adult vector competence for ZIKV (Alomar et al., 2021). Old female *Ae. albopictus* exposed to bifenthrin exhibited an increase in ZIKV disseminated infection rates (Knecht et al., 2018). Collectively, these studies show that insecticide exposure during either larval or adult stages may vary from minimally to substantially enhancing virus infection in mosquitoes.

Although the underlying mechanism responsible for the increase of vector competence in our study is unknown, it is possible that ingestion of the microbial insecticide spinosad in TSB may damage midgut epithelium, thereby diminishing the midgut escape barrier, as observed with larval stages following exposure to spinosad (Fernandes et al., 2019). Another possible mechanism is that the TSB ingestion may change the gut microbiota of adults which may subsequently alter their responses to virus infection (Caragata et al., 2019; Cansado-Utrilla et al., 2021). Other research indicates that exposure to various insecticides affects the bacterial microbiota of mosquito larvae and adults (Tetreau et al., 2018; Arévalo-Cortés et al., 2020; Muturi et al., 2021). Further investigations of the effects of active ingredients in various TSB formulations are warranted.

Our results provide evidence that ingestion of TSB by mosquitoes alters phenotypic responses, including traits related to mosquito fitness, immunity, and vector competence for viruses. These effects of TSB, as a control agent, extend anticipated acute mortality to chronic effects. A reduction in survival of females following the ingestion of TSB is likely to reduce their vectorial capacity for DENV even if their vector competence increases. This observation may mitigate the concerns regarding the enhancement of susceptibility to DENV in treated females. Our work, along with others, emphasizes the need for the assessment of vector control effects on important aspects of vector biology related to the transmission of viruses.

## Data availability statement

The raw data supporting the conclusions of this article will be made available by the authors, without undue reservation.

## Ethics statement

The animal study was reviewed and approved by the University of Florida’s Institutional Biosafety Committee and Institutional Animal Care and Use Committee.

## Author contributions

AA, EW, and BA conceived the study. AA and BE performed the experiments. AA, ZR, EW, and BA analyzed the data and wrote the manuscript. All authors contributed to the article and approved the submitted version.

## Conflict of interest

The authors declare that the research was conducted in the absence of any commercial or financial relationships that could be construed as a potential conflict of interest.

## Publisher’s note

All claims expressed in this article are solely those of the authors and do not necessarily represent those of their affiliated organizations, or those of the publisher, the editors and the reviewers. Any product that may be evaluated in this article, or claim that may be made by its manufacturer, is not guaranteed or endorsed by the publisher.
